# Supplementary material for: Microbial Diversity and Toxin Risk in Tropical Freshwater Reservoirs of Cape Verde
Source: Toxins (Basel). 2018 May 5;10(5):186. doi: 10.3390/toxins10050186 (PMC5983242; doi:10.3390/toxins10050186)
Supplement: Supplementary file 1 [file toxins-10-00186-s001.pdf]

Supplementary Materials: Microbial Diversity and Toxin Risk in Tropical Freshwater Reservoirs of Cape Verde

Ana P. Semedo-Aguiar, Jose B. Pereira-Leal and Ricardo B. Leite

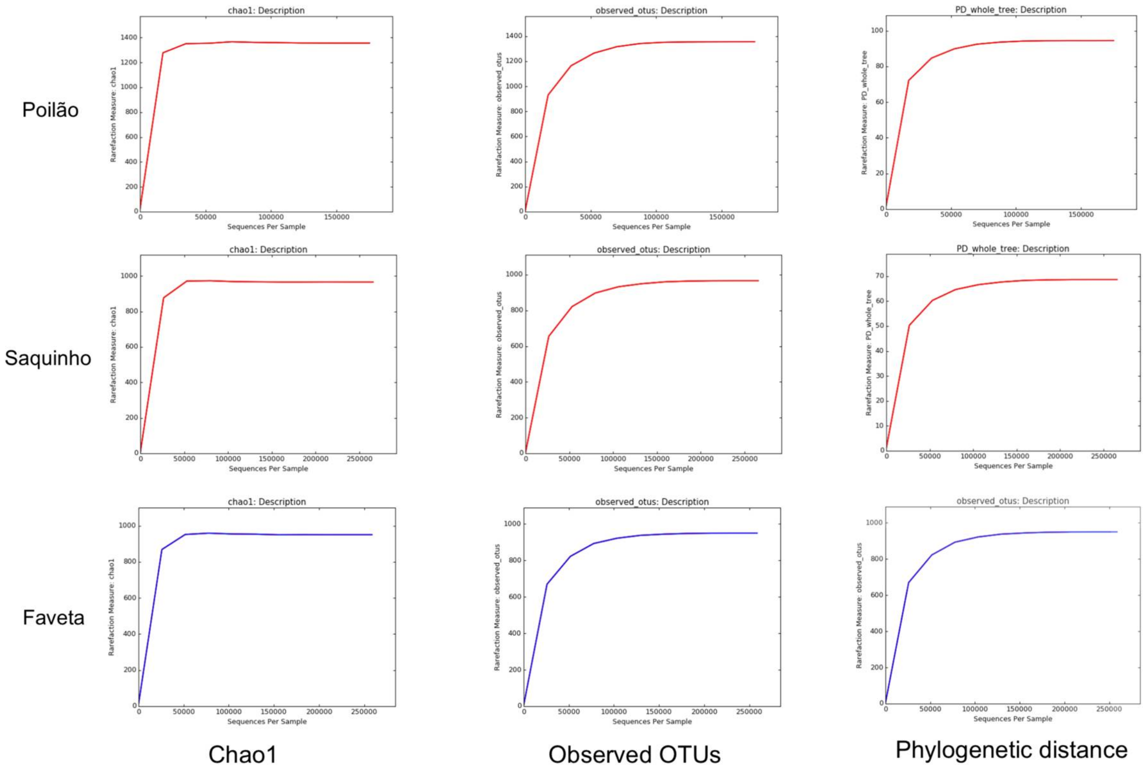

Figure S1. Rarefaction curves per number of reads for each sampled reservoir. Representation based in Chao1, Observed OTUs and Phylogenetic distance whole tree of the 16sRNA gene amplicons of Illumina sequencing.

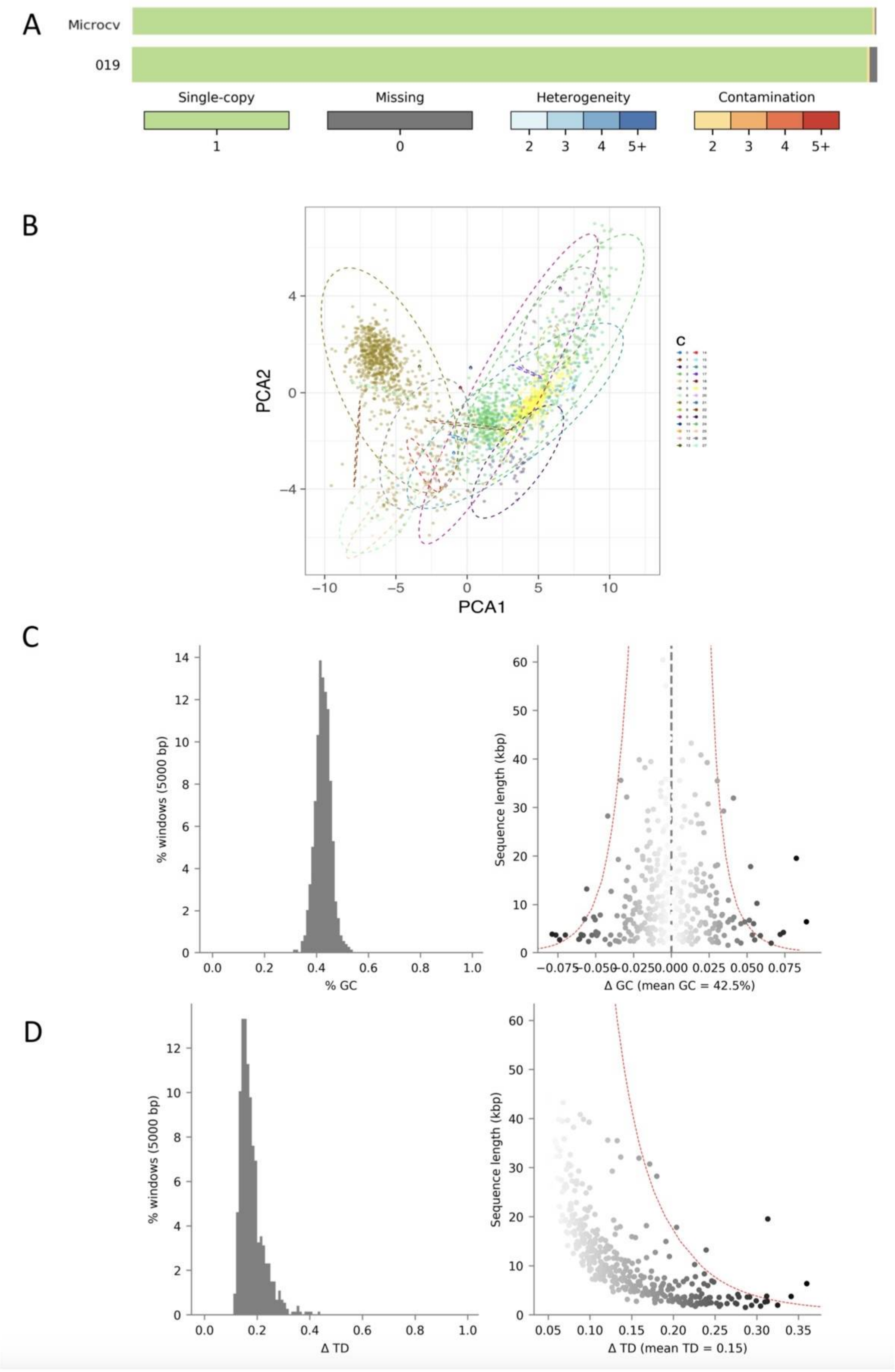

Figure S2. Faveta reservoir. Degree of completeness and contamination of the *M.* cf. *aeruginosa* CV01 bin detected using CONCOCT and assembly produced by SPAdes (panel A), Graphical representation of a PCA of clusters based on composition and coverage (bin 19 in yellow). Left Panel C --- Histogram of GC distribution of sequences within bin 19, Right Panel C --- Deviation of the average of the entire assembly, x--- axis represents GC and y---axis the size. Left Panel D --- Histogram of Manhattan distance between tetranucleotide signature of bin 19 and the entire assembly, Right Panel D --- Deviation of the average of the entire assembly, x axis represents GC and y axis the size.

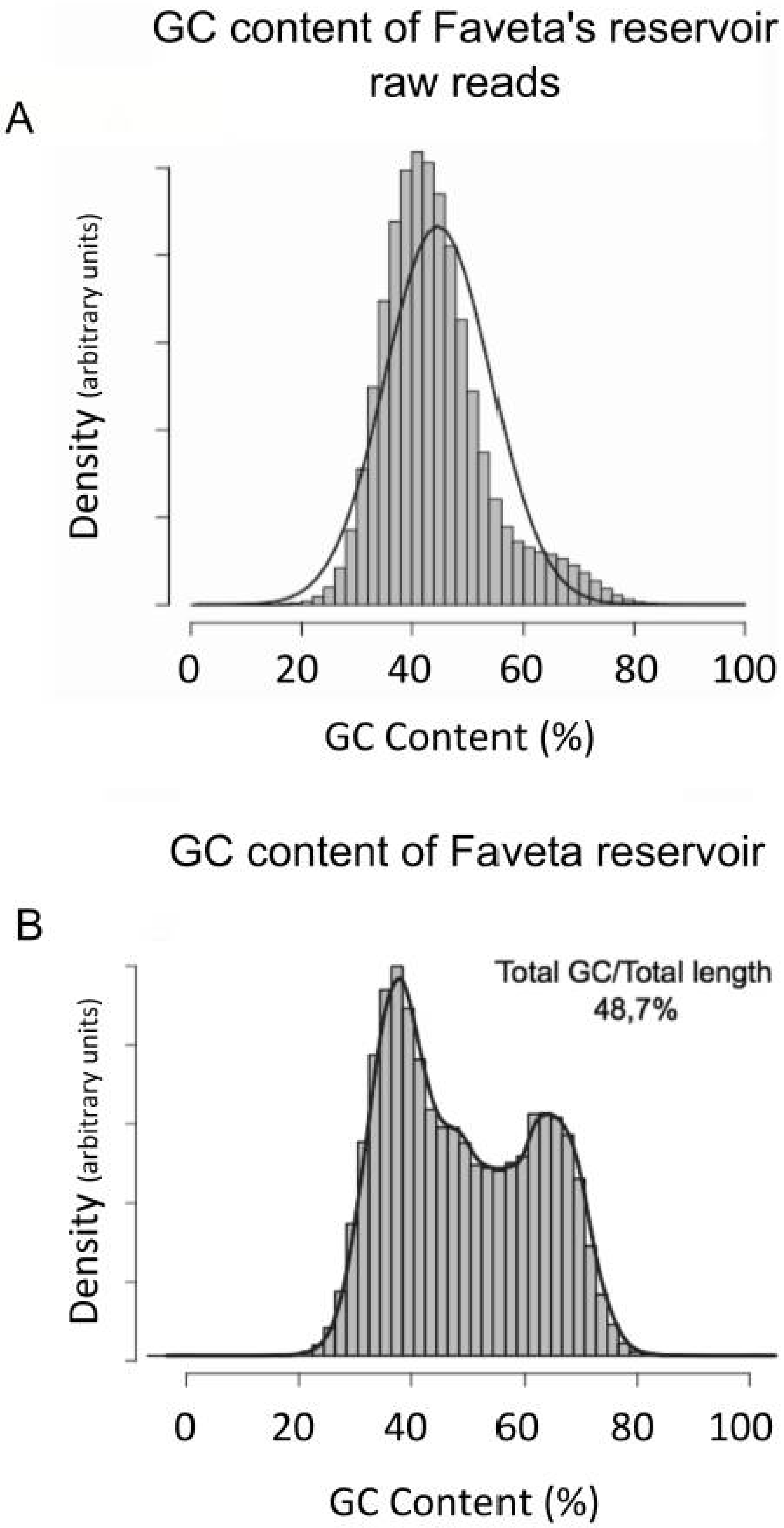

Figure S3. The GC content of the raw reads and the final assembled metagenome from Faveta reservoir. GC content of the raw reads and of the assembled genomes in the histograms of panels A and B, respectively.

Table S1. Degree of completeness and contamination and Percentage of Mapped reads.

Degree of completeness and contamination of CONCOCT bins

Bin Id	Marker lineage	Geno-mes	Mar-kers	marker sets	0	1	2	3	4	5+	Comple-teness	Contami-nation	Strain heterogeneity
all	k__Bacteria (UID203)	5449	103	58	0	34	35	22	10	2	100.00	69.47	1.66
19	p_Cyanobacteria (UID2192)	79	582	456	6	574	2	0	0	0	98.79	0.15	0.00
24	o_Actinomycetales (UID1696)	455	315	190	230	83	2	0	0	0	23.96	0.60	50.00
7	o_Rhodospirillales (UID3754)	63	336	201	259	71	6	0	0	0	22.38	1.52	16.67
3	k_Bacteria (UID1453)	901	171	117	120	51	0	0	0	0	15.68	0.00	0.00
16	k_Bacteria (UID203)	5449	103	58	102	1	0	0	0	0	1.72	0.00	0.00
26	k_Bacteria (UID203)	5449	104	58	102	2	0	0	0	0	0.31	0.00	0.00
27	root (UID1)	5656	56	24	56	0	0	0	0	0	0.00	0.00	0.00
25	root (UID1)	5656	56	24	56	0	0	0	0	0	0.00	0.00	0.00
23	root (UID1)	5656	56	24	56	0	0	0	0	0	0.00	0.00	0.00
22	root (UID1)	5656	56	24	56	0	0	0	0	0	0.00	0.00	0.00
21	root (UID1)	5656	56	24	56	0	0	0	0	0	0.00	0.00	0.00
20	root (UID1)	5656	56	24	56	0	0	0	0	0	0.00	0.00	0.00
18	root (UID1)	5656	56	24	56	0	0	0	0	0	0.00	0.00	0.00
17	root (UID1)	5656	56	24	56	0	0	0	0	0	0.00	0.00	0.00
15	root (UID1)	5656	56	24	56	0	0	0	0	0	0.00	0.00	0.00
14	root (UID1)	5656	56	24	56	0	0	0	0	0	0.00	0.00	0.00
13	root (UID1)	5656	56	24	56	0	0	0	0	0	0.00	0.00	0.00
12	root (UID1)	5656	56	24	56	0	0	0	0	0	0.00	0.00	0.00
11	root (UID1)	5656	56	24	56	0	0	0	0	0	0.00	0.00	0.00
10	root (UID1)	5656	56	24	56	0	0	0	0	0	0.00	0.00	0.00
9	root (UID1)	5656	56	24	56	0	0	0	0	0	0.00	0.00	0.00
8	root (UID1)	5656	56	24	56	0	0	0	0	0	0.00	0.00	0.00
6	root (UID1)	5656	56	24	56	0	0	0	0	0	0.00	0.00	0.00
5	root (UID1)	5656	56	24	56	0	0	0	0	0	0.00	0.00	0.00
4	root (UID1)	5656	56	24	56	0	0	0	0	0	0.00	0.00	0.00
2	root (UID1)	5656	56	24	56	0	0	0	0	0	0.00	0.00	0.00
1	root (UID1)	5656	56	24	56	0	0	0	0	0	0.00	0.00	0.00
0	root (UID1)	5656	56	24	56	0	0	0	0	0	0.00	0.00	0.00

Mapped reads to per bin

Bin Id	Bin size (Mbp)	aln.sorted.markdup.sorted: mapped reads	aln.sorted.markdup.sorted: % mapped reads	aln.sorted.markdup.sorted: % binned populations	aln.sorted.markdup.sorted: % community
0	0.00	263	0.01	5.92	5.70
1	0.00	32	0.00	0.32	0.31
2	0.06	945	0.04	0.54	0.52
3	0.42	7529	0.33	0.57	0.54
4	0.00	28	0.00	0.54	0.52
5	0.00	78	0.00	0.50	0.48
6	0.00	61	0.00	0.55	0.53
7	1.25	16924	0.75	0.43	0.42
8	0.01	4751	0.21	12.25	11.80
9	0.00	71	0.00	0.50	0.49
10	0.00	22	0.00	0.50	0.48
11	0.00	681	0.03	19.35	18.63
12	0.00	723	0.03	17.93	17.27
13	0.00	18	0.00	0.54	0.52
14	0.01	66	0.00	0.40	0.39
15	0.00	838	0.04	12.25	11.79
16	0.37	77707	3.42	6.70	6.45
17	0.00	65	0.00	0.58	0.56
18	0.00	23	0.00	0.34	0.33
19	4.61	2069173	91.14	14.29	13.76
20	0.00	51	0.00	0.75	0.72
21	0.00	112	0.00	0.92	0.89
22	0.00	100	0.00	1.39	1.34
23	0.00	9	0.00	0.27	0.26
24	0.38	4855	0.21	0.41	0.39
25	0.01	107	0.00	0.46	0.44
26	0.03	392	0.02	0.40	0.39
27	0.03	367	0.02	0.40	0.38
unbinned	4.43	84293	3.71	NA	3.71

Degree of completeness and contamination of Spades assembly

Bin Id	Marker lineage	Geno-mes	Mar-kers	marker sets	0	1	2	3	4	5+	Comple-teness	Contami-nation	Strain heterogeneity
1	p_Cyanobacteria (UID2192)	79	582	456	1	579	2	0	0	0	99.89	0.15	0.00

Mapped reads from the SPADES assembly

**Bin Id**	**Bin size (Mbp)**	**aln.sorted.markdup.sorted: mapped reads**	**aln.sorted.markdup.sorted: % mapped reads**	**aln.sorted.markdup.sorted: % binned populations**	**aln.sorted.markdup.sorted: % community**
0	4.97	2297922	100	100	100

Table S2. KEGG functional annotation of the predicted transcriptome of *Microcystis cf. aeruginosa* CV01.

KEGG function categories		Value
Carbohydrate metabolism	Metabolism	203
Energy metabolism	Metabolism	182
Lipid metabolism	Metabolism	40
Nucleotide metabolism	Metabolism	82
Amino acid metabolism	Metabolism	169
Metabolism of other amino acids	Metabolism	35
Glycan biosynthesis and metabolism	Metabolism	24
Metabolism of cofactors and vitamins	Metabolism	139
Metabolism of terpenoids and polyketides	Metabolism	36
Biosynthesis of other secondary metabolites	Metabolism	25
Xenobiotics biodegradation and metabolism	Metabolism	26
Transcription	Genetic Information Processing	4
Translation	Genetic Information Processing	80
Folding, sorting and degradation	Genetic Information Processing	37
Replication and repair	Genetic Information Processing	56
Membrane transport	Environmental Information Processing	76
Signal transduction	Environmental Information Processing	42
Transport and catabolism	Environmental Information Processing	3
Cell growth and death	Cellular Processes	14
Immune system	Organismal Systems	2
Endocrine system	Organismal Systems	14
Digestive system	Organismal Systems	2
Excretory system	Organismal Systems	1
Nervous system	Organismal Systems	7
Aging	Organismal Systems	8
Environmental adaptation	Organismal Systems	1
Other functions		86
Total protein coding genes with function prediction		1394
Proteins with no KEGG Orthology assignment		3233

Table S3. COG functional categories of the predicted transcriptome of *Microcystis cf. aeruginosa* CV01.

**Number of genes associated with general COG functional categories.**			
**Code**	**Value**	**%age**	**Description**
J	197	4,88	Translation, ribosomal structure and biogenesis
A	0	0,00	RNA processing and modification
K	289	7,71	Transcription
L	391	8,41	Replication, recombination and repair
B	0	0,02	Chromatin structure and dynamics
D	112	2,74	Cell cycle control, Cell division, chromosome partitioning
V	173	3,70	Defense mechanisms
T	340	9,08	Signal transduction mechanisms
M	372	9,61	Cell wall/membrane biogenesis
N	49	1,78	Cell motility
U	111	2,91	Intracellular trafficking and secretion
O	326	8,20	Posttranslational modification, protein turnover, chaperones
C	344	9,87	Energy production and conversion
G	235	6,98	Carbohydrate transport and metabolism
E	386	11,02	Amino acid transport and metabolism
F	117	3,00	Nucleotide transport and metabolism
H	326	8,82	Coenzyme transport and metabolism
I	137	3,83	Lipid transport and metabolism
P	334	10,23	Inorganic ion transport and metabolism
Q	228	5,91	Secondary metabolites biosynthesis, transport and catabolism
R	0	0,00	General function prediction only
S	2679	57,34	Function unknown
-	-		Not in COGs
